# Sex-related differences in survival based on [^18^F]FDG PET/CT metabolic tumor volume after liver transplantation for colorectal liver metastases

**DOI:** 10.3389/ti.2026.16406

**Published:** 2026-06-16

**Authors:** Nadide Mutlukoca Stern, Svein Dueland, Pål-Dag Line, Trygve Syversveen, Harald Grut

**Affiliations:** 1 Department of Radiology, Vestre Viken Hospital Trust, Drammen, Norway; 2 Department of Radiology and Nuclear Medicine, Oslo University Hospital, Oslo, Norway; 3 Institute of Clinical Medicine, University of Oslo, Oslo, Norway; 4 Department of Transplantation Medicine, Oslo University Hospital, Oslo, Norway; 5 Nuclear Medicine and Radiation Biology Research Group, Department of Clinical Medicine, UiT The Arctic University of Norway, Tromsø, Norway

**Keywords:** [18F]FDG PET/CT, colorectal liver metastases, liver transplantation, metabolic tumor volume, sex-related differences

## Abstract

[^18^F]FDG PET/CT-derived metabolic tumor volume (MTV) is a strong prognostic biomarker in patients undergoing liver transplantation (LT) for non-resectable colorectal liver metastases (nCRLM). This study aimed to evaluate whether MTV-based survival outcomes after LT for nCRLM differ between males and females. Patients who underwent LT for nCRLM between 2006 and 2022 were included (n = 45). Baseline characteristics were compared using the Mann–Whitney U test and Fisher’s exact test. Overall survival (OS), disease-free survival (DFS), and survival after relapse (SAR) were estimated using Kaplan–Meier analysis with the log-rank test. Cox regression analyses including sex, dichotomized MTV, and an interaction term between sex and MTV were performed. Female patients had significantly shorter OS compared with male patients (median 58 vs. 92 months, *p* = 0.047). Low MTV was associated with significantly longer OS, DFS and SAR in both sexes. Among patients with high MTV, females had significantly shorter OS than males (median 25 vs. 59 months, p < 0.001). Females had more non-pulmonary recurrences than males (*p* = 0.006) and shorter median SAR than males (17 vs. 46 months, p < 0.001) with high MTV. A significant interaction between sex and MTV was observed, indicating a particularly adverse prognostic impact of high MTV in female patients.

## Graphical Abstract

**Figure:**
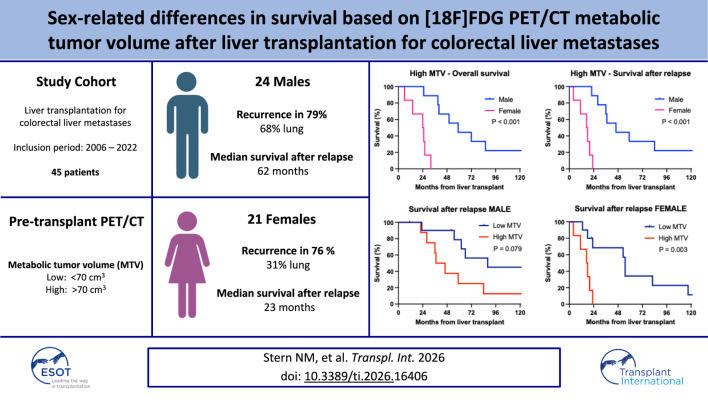

## Introduction

Liver transplantation (LT) for non-resectable colorectal liver metastases (nCRLM) has evolved from an experimental approach to a potentially curative treatment in a highly selected group of patients with liver-limited disease [1]. The pioneering SECA study from Oslo University Hospital demonstrated long-term overall survival (OS) in patients transplanted beyond traditional resection criteria, thereby establishing LT as a viable oncological surgical strategy [2, 3]. Comparable outcomes have subsequently been reported from other centers, including the Rochester protocol in the United States [4], demonstrating the reproducibility of this concept in independent cohorts.

Accurate patient selection remains critical, as disease recurrence occurs in most cases. Conventional morphologic and biochemical markers alone provide only an indirect approximation of tumor biology or behaviour, as they rely on surrogate measures such as carcinoembryonic antigen levels, tumor growth rate, response to chemotherapy, and the presence or absence of extrahepatic disease.

[^18^F]FDG PET/CT provides quantitative information on tumor glucose metabolism, which may reflect tumor aggressiveness in addition to providing the well-established volumetric parameter, metabolic tumor volume (MTV), for quantifying metabolic burden and predicting prognosis in colorectal cancer [5]. In a seminal study, Grut et al. [6] demonstrated that pre-transplant MTV was a strong predictor of survival after LT in nCRLM. These findings were subsequently confirmed in a larger national cohort [7] and validated externally by Wehrle et al. [8], thereby establishing MTV as a reproducible prognostic biomarker in transplant oncology [9].

Recently, the TRANSMET trial [10] demonstrated that patients randomized to LT plus chemotherapy against chemotherapy alone obtained a substantial transplant benefit thereby providing level 1 evidence for LT in nCRLM. In a recent multicentre study, Vitale et al. identified female sex as an independent predictor of inferior survival after LT for nCRLM, despite otherwise comparable clinicopathologic features [11]. MTV was not part of the risk stratification in this study.

The aim of this study was therefore to explore potential sex-related differences in the prognostic value of pre-transplant MTV measured by [^18^F]FDG PET/CT.

## Materials and methods

### Patient selection

This retrospective cohort included consecutive patients who underwent LT for nonresectable liver-only CRLM at Oslo University Hospital, Norway, between 2006 and 2022 (SECA 1 and 2 studies). All patients had a histologically confirmed colorectal adenocarcinoma that had been surgically resected and were evaluated by a multidisciplinary hepatobiliary tumor board prior to inclusion. Eligibility criteria included R0 resection of the primary tumor, nCRLM confined to the liver, absence of extrahepatic disease on CT, MRI or [^18^F]FDG PET/CT, an Eastern Cooperative Oncology Group performance status of 0–1, body mass index <30 and having received pre-transplant chemotherapy.

### [^18^F]FDG PET/CT procedure and MTV analysis

All SECA patients underwent a standard routine whole-body PET/CT examination prior to LT. Any chemotherapy treatment was paused for 4–6 weeks before the PET/CT scan. Patients fasted for at least 6 h, and plasma glucose levels were measured before intravenous injection of [^18^F]FDG. Imaging was performed approximately 60 min post-injection with image acquisition from the skull base to mid-thigh, on hybrid PET/CT scanners (Siemens Biograph 64/16, or GE Healthcare Discovery MI), using iterative reconstruction with attenuation and scatter correction. For anatomical correlation and attenuation correction, a low-dose CT without intravenous contrast enhancement was performed. Metabolic tumor volumes were delineated semi-automatically using a fixed 40% SUV_max_ threshold with Siemens syngo.via software. Total MTV for each patient was calculated as the sum of all hepatic MTVs.

### Statistical analysis

Continuous variables were reported as medians and compared using the Mann–Whitney U-test while categorical variables were compared using the Fisher’s exact test. Overall survival (OS), disease-free survival (DFS), and survival after relapse (SAR) were estimated using Kaplan–Meier method and compared using the log-rank test. DFS was defined as the time from LT to detection of metastatic lesions, local relapse identified by CT/MRI or PET, detection of new CRC primary, or other malignant disease. OS was defined as the time from LT to death or end of follow-up. SAR was defined as the time from recurrence to death. MTV was analysed as a dichotomized variable using a predefined cut-off of 70 cm^3^ (low ≤70 cm^3^, high >70 cm^3^). Cox proportional hazards regression analyses were performed for OS including sex, dichotomized MTV, and an interaction term between sex and MTV to assess whether the prognostic impact of high MTV differed according to sex. The interaction term specifically evaluated whether female patients with high MTV had a different risk profile than expected from the independent effects of sex and MTV alone. Optimal MTV cut-off values for predicting 5-year OS in male and female patients were determined using receiver operating characteristic (ROC) analysis. All statistical analyses were performed using IBM SPSS Statistics version 29 (IBM Corp., Armonk, NY, USA). A P-value <0.05 was considered statistically significant. Kaplan-Meier survival plots were generated using GraphPad Prism version 10.

### Ethics

The study complied with the Declaration of Helsinki, was approved by the Regional Committee for Medical and Health Research Ethics and was registered at ClinicalTrials.gov (NCT01311453 for SECA-1 and NCT01479608 for SECA-2). Written informed consent had been obtained from all participants prior to inclusion in the original studies.

## Results

### Baseline characteristics

The study included 45 patients: 24 (53%) male and 21 (47%) female. Baseline characteristics stratified by sex are summarised in Table 1. Male and female patients had similar age, primary tumor location, MTV, number and size of liver metastases, T and N stage, carcinoembryonic antigen levels, lines of chemotherapy, time of detection of liver metastases and number of patients with progression at LT.

**TABLE 1:** Comparison of baseline characteristics.

Characteristics	Men	Women	P-value
Number of patients	24	21	​
Age at LT, years*	56 (34–71)	58 (44–66)	0.802
Site of primary tumor	​	​	0.658
Left sigmoid and rectum	21	18	​
Right	2	3	
Metabolic tumor volume, cm^3^ *	27.54 (0–397.10)	14.44 (0–874.12)	0.475
Number of metastases at LT*	7.5 (1–40)	7 (2–53)	0.464
Size of the largest metastasis at LT, mm*	35.5 (7–130)	28 (3–120)	0.241
CEA (ng/mL)*	7.25 (1–2002)	2 (1–404)	0.070
Time of liver metastases	​	​	1.000
Synchronous	21 (88)	19 (91)	​
Metachronous	3 (12)	2 (9)	​
Progression at LT	​	​	1.000
Yes	9 (37)	7 (33)	​
No	15 (63)	14 (67)	​
Lines of chemotherapy	​	​	0.197
1	11 (46)	11 (52)	​
2	9 (38)	10 (48)	​
3	4 (16)	0	​
First site relapse, n (%)	​	​	**0.006**
Lungs	13 (68)	5 (31)	​
Non-lung	6 (32)	11 (69)	
T stage, n (%)	​	​	0.177
(y)pT0	0	3 (14)	​
(y)pT1	0	1 (5)	
(y)pT2	3 (13)	2 (8)	
(y)pT3	20 (83)	15 (71)	
(y)pT4	1 (4)	0	
N Stage, n (%)	​	​	0.675
(y)pN0	12 (50)	9 (43)	​
(y)pN1	7 (29)	9 (43)	
(y)pN2	5 (21)	3 (14)	
KRAS status	​	​	0.890
Wild type	17 (71)	14 (67)	​
Mutated	6 (25)	5 (24)	​
Unknown	1 (4)	2 (9)	​

* Median (range).

^+^ Increased size of liver metastases on CT, and/or increased carcinoembryonic antigen (CEA).

LT, liver transplantation.

KRAS, kirsten rat sarcoma viral oncogene homolog.

### Survival analysis

#### Overall survival

**FIGURE 1:**
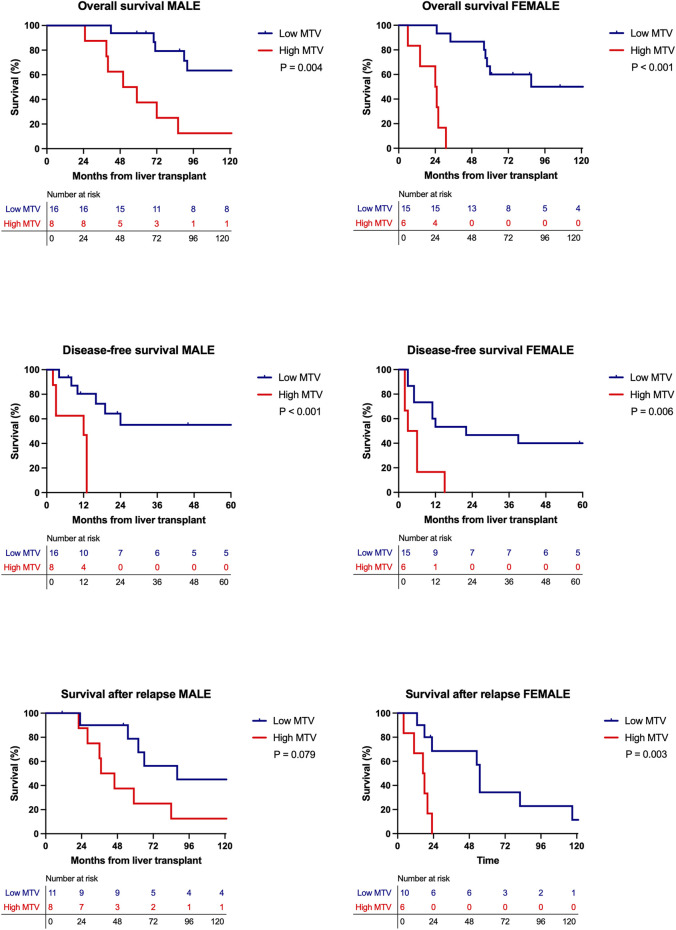
Kaplan-Meier survival curves for patients with high versus low metabolic tumor volume for males and females. Patients with low metabolic tumor volume had significantly longer overall survival and disease-free survival in both sexes and female patients had significantly shorter survival after relapse.

**FIGURE 2:**
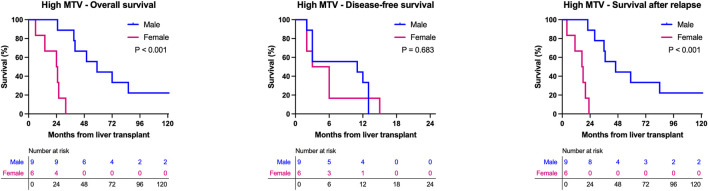
Kaplan-Meier survival curves for patients with high metabolic tumor volume comparing male and female patients. Males had significantly longer overall survival and survival after relapse than females.

Median OS was 92 months in male patients and 58 months in female patients (p = 0.047, log-rank test). Males with low MTV had a median OS of 166 months compared to 59 months in males with high MTV (p = 0.004). Females with low MTV had an OS of 87 months compared to 24 months in females with high MTV (p < 0.001) (Figure 1). Overall survival did not differ significantly between males and females with low MTV (median 166 vs. 87 months, p = 0.053). Among patients with high MTV, males had significantly longer OS than females (median 59 months vs. 24 months, p < 0.001) (Figure 2).

In Cox regression analyses for OS, high MTV was associated with increased risk of death in both univariable (HR 4.64, 95% CI 2.15–10.01, p < 0.001) and multivariable analyses (HR 4.68, 95% CI 1.55–14.15, p = 0.006). Sex showed a non-significant association with OS in univariable analysis (HR 2.13, 95% CI 0.99–4.58, p = 0.053) and multivariable analysis (HR 2.43, 95% CI 0.85–6.95, p = 0.098). A significant interaction between sex and MTV was observed (HR 8.20, 95% CI 1.12–60.11, p = 0.038) (Table 2). This interaction term reflects that female patients with high MTV had particularly poor survival beyond the independent effects of sex and MTV alone.

**TABLE 2:** Cox regression analyses for overall survival.

Variable	Univariable HR (95% CI)	P-value	Multivariable HR (95% CI)	P-value
Sex (male/female)	2.13 (0.99–4.58)	0.053	2.43 (0.85–6.95)	0.098
MTV (high/low)	4.64 (2.15–10.01)	**<0.001**	4.68 (1.55–14.15)	**0.006**
Interaction (MTV × sex)	-	-	8.20 (1.12–60.11)	**0.038**

Receiver operating characteristic (ROC) analysis identified optimal MTV cut-off values for predicting 5-year OS of 76 cm^3^ for females and 66 cm^3^ for males. The area under the curve was 0.74 for both sexes.

#### Disease-free survival

Among male patients with low MTV, median DFS was not reached. In contrast, males with high MTV had median DFS of 12 months (p < 0.001). Female patients with low MTV had an estimated DFS of 22 months compared with 3 months in those with high MTV (p = 0.006) (Figure 1). All patients with high MTV experienced recurrence, however, no significant difference in DFS between sexes (median 11 vs. 3 months, p = 0.683) (Figure 2). Female patients had significantly more non-pulmonary relapses than males (p = 0.006).

#### Survival after relapse

Male patients with low MTV had a median SAR of 88 months compared with 46 months in those with high MTV (p = 0.079). Female patients with low MTV had a median SAR of 55 months compared with 17 months in those with high MTV (p = 0.003) (Figure 1). Among patients with high MTV, males had significantly longer SAR than females (median 46 vs. 17 months, p < 0.001) (Figure 2).

## Discussion

This study confirms that [^18^F] FDG PET/CT-derived metabolic tumor volume is a robust predictor of survival following LT for nCRLM and demonstrates a significant interaction between sex and MTV, indicating that the adverse prognostic impact of high MTV (>70 cm^3^) differs between males and females. Female recipients appear to have a lower tolerance for high metabolic tumor burden, with more frequent non-pulmonary recurrences, resulting in poorer outcomes despite otherwise similar baseline characteristics.

The prognostic importance of MTV has been consistently demonstrated in prior work from our group [8, 9] and subsequently validated internationally [10]. The present findings expand this evidence by formally demonstrating a sex-dependent interaction between MTV and survival in multivariable analyses. In a recent multicentre cohort, not including PET-derived MTV data, Vitale et al. reported that female sex was independently associated with poorer outcomes after LT [11].

Sexual dimorphism in cancer is based on the observation that differences in the incidence and mortality of non sex-related cancers cannot be explained solely by sociobehavioral (gender related) factors, but may instead be related to biological differences, including sex chromosomes [12]. Variations in health-seeking behavior, lifestyle factors, and environmental exposures may contribute to these disparities, but are insufficient to explain the full extent of sex-related differences observed in cancer epidemiology [13]. For most cancers unrelated to reproductive function, both incidence and prevalence tend to be modestly higher in males than in females, with the exceptions of thyroid and gallbladder cancer. Similarly, cancer-related mortality is generally higher in males than in females [13, 14].

Sex hormones may play a role in the initiation and progression of CRC, with age-dependent effects. While premenopausal females generally exhibit superior survival compared with age-matched males, post-menopausal females often experience poorer outcomes, potentially due to diagnosis at more advanced stages or intrinsically more aggressive tumor characteristics, such as right-sided tumor location and BRAF mutations [11, 15]. Sex-specific patterns of adipose distribution and lipid metabolism may further influence cancer risk. Visceral fat-associated inflammation is a recognized driver of carcinogenesis and may contribute to the increased incidence of several cancers after menopause, when females tend to shift towards greater visceral adiposity [13].

The female immune system appears to have a higher activation threshold for T-cell mediated immune responses, partly driven by estrogen, potentially reflecting immunological adaptations related to pregnancy [16]. Sex-related differences regarding immune system responses have been shown to influence disease onset, symptom presentation, patterns of progression and treatment outcomes across a range of conditions, including autoimmune disorders, infectious diseases, cancers and vaccine efficacy [17]. Also, the liver is an estrogen-sensitive organ [18], and experimental data suggest that estrogen may modulate the microenvironment of liver metastases in a sex-specific manner [19]. However, these proposed mechanisms remain speculative and are not directly supported by data in the present study.

Interestingly, population studies have shown that females are about 25% less likely to undergo surgery for liver metastases, and in this cohort, the all-cause mortality is significantly higher in females compared to males. Since the LT patients all had nCRLM, this may indicate that the gender related effects on outcome are related to biological differences in tumor biological behavior [20].

Given a limited availability of liver grafts, optimizing patient selection is of clear clinical relevance. In the present study, males more frequently presented with pulmonary metastases (two-thirds of recurrences), which are known to have relatively indolent course and are often amenable to pulmonary resection with prolonged SAR [21, 22]. In contrast, approximately two-thirds of females experienced non-pulmonary recurrence, a pattern associated with poorer survival outcomes [22].

The current analysis shows that males with high MTV had an acceptable median OS of 59 months. Thus, males who otherwise demonstrate favorable characteristics during pre-transplant evaluation may still be considered for inclusion despite high MTV (>70 cm^3^), although this should be interpreted with caution given the limited sample size. The ROC analysis identified optimal MTV cut-off values for predicting 5-year OS of 76 cm^3^ in females and 66 cm^3^ in males. Accordingly, the currently used, non-sex-specific cut-off value of 70 cm^3^ appears appropriate, with no clear indication at present for separate MTV thresholds for males and females.

Several limitations merit consideration. This was a retrospective study with a limited sample size, reflecting the rarity of LT for nCRLM; consequently, the statistical power for sex-stratified analyses should be regarded as modest. Exact information on pre- and postmenopausal status among female patients was unavailable. However, given the median age of the female patients was 58 years and that all patients received chemotherapy, it is likely that a majority were postmenopausal at the time of LT. PET/CT acquisition protocols and scanner technology evolved during the inclusion period, which may have introduced variability in SUV quantification, although this is unlikely to have substantially affected MTV measurements. The 70 cm^3^ MTV threshold is empirically derived and should not be considered as an absolute. Minor variations in preoperative chemotherapy and immunosuppressive regimens may also have influenced outcomes. Finally, as with all observational studies, unmeasured confounding factors cannot be excluded. Nonetheless, the use of uniform national selection criteria and long-term follow-up adds robustness to the observed associations.

Introducing different inclusion and exclusion criteria based on sex would be premature and is not the intention of this study. Some recent papers, however, indicate that relevant gender specific differences are clinically relevant [20] and the findings from the current study underscore that biological sex should be considered as one of several factors when planning and individualizing treatment strategies aimed at achieving the best possible outcomes.

In summary, pre-transplant [^18^F]FDG PET/CT-derived MTV remains a robust prognostic biomarker after LT for nCRLM. Female patients exhibit poorer OS and SAR at high MTV levels. These findings suggest that sex may modify the prognostic impact of MTV but should be considered hypothesis-generating and require validation in larger cohorts. The underlying mechanisms remain to be elucidated.

## Conclusion

Female patients had shorter OS than males, including when the analysis was restricted to patients with high MTV. This may be explained by the post-transplant recurrence patterns, with females developing more unfavourable non-pulmonary recurrences. These findings are hypothesis-generating and should be interpreted with caution and require validation in larger cohorts before clinical implications can be drawn.

## Data Availability

The datasets presented in this article are not readily available because of regulatory restrictions on data sharing but are available from the corresponding author upon reasonable request. Requests to access the datasets should be directed to Harald Grut, harald.grut@gmail.com.
